# Esophageal metastasis of renal cancer 10 years after nephrectomy

**DOI:** 10.1007/s10388-013-0411-7

**Published:** 2014-01-08

**Authors:** Wataru Izumo, Masaho Ota, Kosuke Narumiya, Yushi Shirai, Kenji Kudo, Masakazu Yamamoto

**Affiliations:** Department of Surgery, Institute of Gastroenterology, Tokyo Women’s Medical University, 8-1, Kawada-cho, Shinjuku-ku, Tokyo, 162-8666 Japan

**Keywords:** Renal cancer, Esophageal metastasis, Surgery

## Abstract

The patient was a 65-year-old man, who had undergone right nephrectomy for renal cancer in 2002. At that time, histopathological examination revealed clear cell carcinoma (pT3a, pN0, M0, and pStage III). Postoperatively, he received natural interferon alpha (6 million units 3 times a week) from November 2002 to February 2005, and showed no evidence of recurrence. However, he noticed dysphagia in March 2012. Endoscopy revealed a pedunculated polypoid tumor in the mid-esophagus and biopsies were taken showing a clear cell carcinoma. Contrast-enhanced thoracoabdominal CT scanning identified a pedunculated polypoid tumor in the mid-thoracic esophagus and enlargement of a lymph node adjacent to the right main bronchus. With a diagnosis of esophageal and lymph node metastases of renal cancer, the patient underwent esophagectomy with right thoracotomy with reconstruction by a posterior mediastinal stomach tube. Postoperative histopathological examination revealed clear cell carcinoma. Because esophageal metastasis of renal cancer is extremely rare, this case is reported here together with discussions of the relevant literature.

## Introduction

Renal cell cancer is likely to hematogenously metastasize to the lung, bone, or liver, but metastasis to the esophagus is very rare. We report a patient with esophageal and mediastinal lymph node metastases that were treated surgically 10 years after the resection of primary renal cancer.

## Case report

Patient: A 65-year-old man.

Presenting complaint: difficulty in swallowing.

### Past history

In 2002, the patient underwent right nephrectomy for renal cancer at another hospital. Histopathological examination revealed clear cell carcinoma (G2 > G3, pT3a, pN0, M0, and pStage III). Postoperative adjuvant therapy was performed with natural interferon alpha (Dainippon Sumitomo Pharma, Osaka, Japan) at a dose of 6 million units 3 times a week from 2002 to 2005, with no evidence of recurrence.

Present illness: in March 2012, the patient complained of dysphagia. Because upper gastrointestinal endoscopy detected a pedunculated polypoid tumor in the mid-esophagus and biopsy revealed clear cell carcinoma, he was referred to our hospital with a diagnosis of esophageal metastasis of renal cancer.

Findings on admission: the patient was 159 cm tall and weighed 51.8 kg. His temperature was 36.6 °C, blood pressure was 108/60 mmHg, and pulse rate was 77/min (regular). There was an upper abdominal scar from the transverse incision for his old right nephrectomy.

Hematology and biochemistry tests: there were no abnormal findings (Table 1).

**Table 1:** Examination on admission

WBC: 6,460/μl	Alb: 4.1 g/dl
RBC: 417 × 10^4^/μl	T-bil: 0.5 mg/dl
Hb: 13.3 g/dl	AST: 15 IU/l
Ht: 39.8 %	ALT: 17 IU/l
Plt: 23.4 × 10^4^/μl	ALP: 247 IU/l
CEA: 3.2	γ-GTP: 42 IU/l
CA19-9: 1.2	BUN: 15.5 mg/dl
PT: 79.1 %	Cr: 1.05 mg/dl
PT(INR): 1.08	CRP: 0.63 mg/dl

**Fig. 1:**
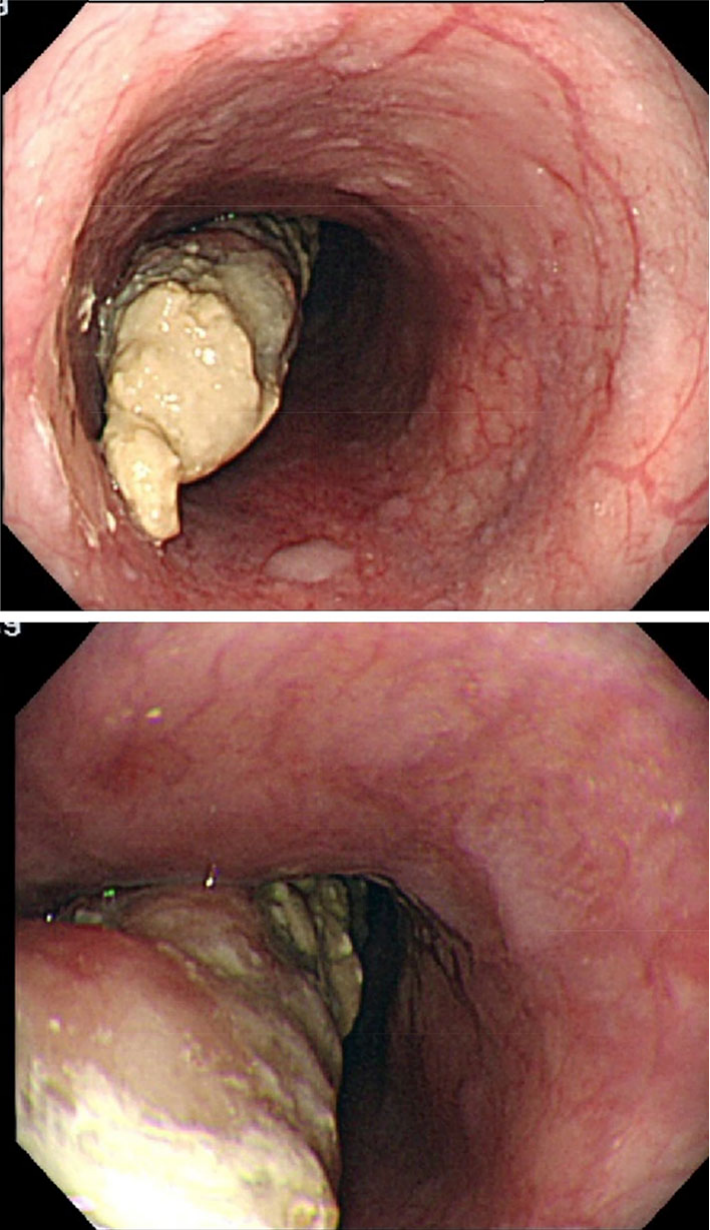
Upper gastrointestinal endoscopy shows a type 0-Ip pedunculated tumor on the anterior wall at 29–35 cm from the incisors

Upper gastrointestinal endoscopy: a pedunculated polypoid tumor was noted on the anterior wall of the esophagus at 29–35 cm from the incisors (Fig. 1). Biopsy revealed clear cell carcinoma. The tumor showed invasion for submucosal layer on endoscopic ultrasonography.

**Fig. 2:**
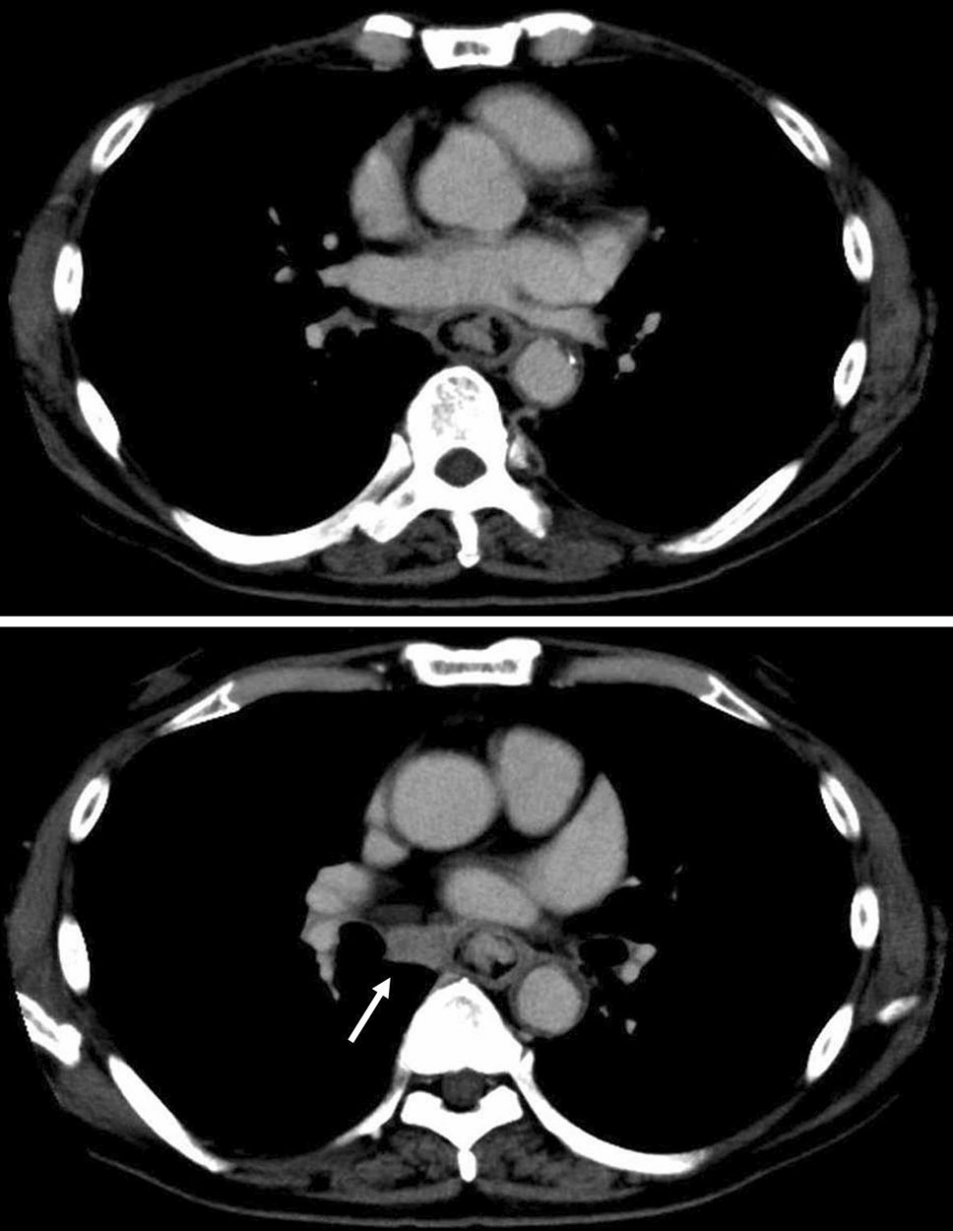
Contrast-enhanced thoracoabdominal CT reveals a tumor in the mid-esophagus, and an enlarged lymph node adjacent to the right main bronchus

Thoracoabdominal CT: A a tumor was seen in the mid-thoracic esophagus, and a lymph node adjacent to the right main bronchus was enlarged (Fig. 2).

**Fig. 3:**
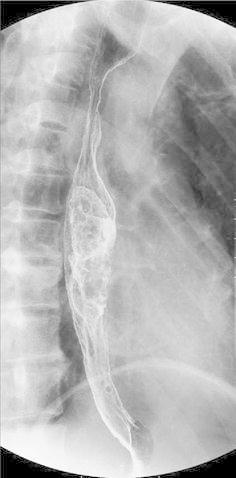
Upper gastrointestinal contrast radiography shows a pedunculated tumor in the mid-esophagus

Upper gastrointestinal contrast radiography: a pedunculated polypoid tumor was identified in the mid-thoracic esophagus (Fig. 3).

Positron emission tomography/CT: uptake was noted at the mid-thoracic esophagus and in a lymph node adjacent to the right main bronchus.

Based on these findings, a diagnosis of esophageal metastasis and mediastinal lymph node metastasis of renal cancer was made, and esophagectomy via right thoracotomy was performed with reconstruction using a posterior mediastinal stomach tube. Endoscopic treatment was not indicated for the esophageal metastasis because of its invasion of the submucosal layer and associated obstruction.

### Operation

Surgery was performed under general anesthesia plus epidural anesthesia. A midline incision was made in the upper abdomen and a thin stomach tube was fashioned on the side of the greater curvature. The patient was then placed in the left lateral decubitus position, and thoracotomy was performed at the right 5th intercostal space. The thoracic duct was preserved. After dissecting the azygos vein and right bronchial artery, the esophagus was dissected free and cut at the level of the aortic arch. An enlarged lymph node (right main bronchus lymph node) was adherent to the cartilage of the right main bronchus, so this node was dissected free. Reconstruction was performed by high intramediastinal anastomosis and the operation was completed. The operating time was 6 h and 59 min and the blood loss was 234 ml.

**Fig. 4:**
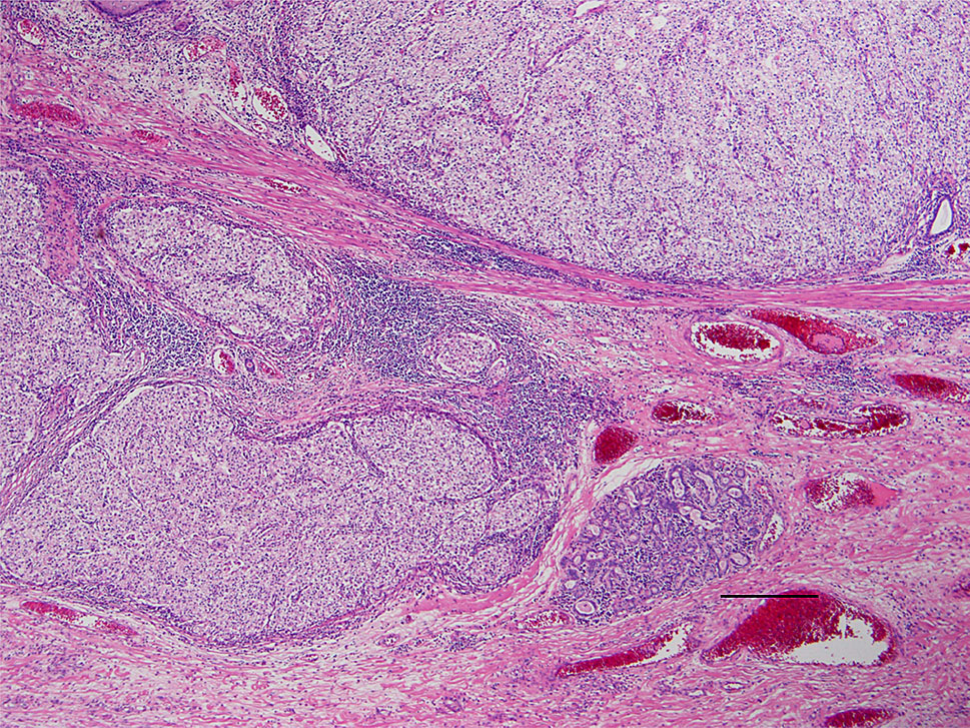
Histopathological findings: small alveolar proliferations of atypical cells with clear cytoplasm can be seen

**Fig. 5:**
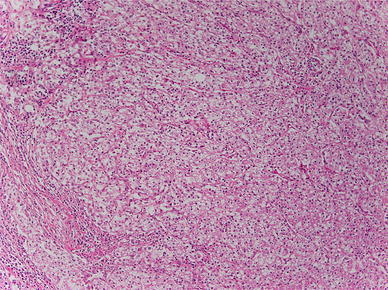
Histopathological findings: substantial proliferation of tumor cells with clear cytoplasm

Histopathological findings: small alveolar proliferations of atypical cells with clear cytoplasm were noted (Fig. 4). The diagnosis was clear cell carcinoma. Because histopathological examination at the time of right nephrectomy in 2002 also revealed proliferation of tumor cells with clear cytoplasm (Fig. 5), metastasis of renal cancer to the esophagus was suggested.

Postoperative course: although suture dehiscence occurred, the patient’s condition improved with conservative treatment (enteral and central venous hyperalimentation) and he was discharged from hospital 27 days postoperatively.

## Discussion

Metastatic esophageal cancer is relatively uncommon. The mechanism of metastasis can be divided into direct invasion into adjacent organs (such as the lungs, trachea/bronchus, thyroid, larynx, hypopharynx, and stomach) or hematogenous/lymphogenous invasion of distant organs such as the uterus or liver. Mizobuchi et al. [1] reviewed the autopsy findings of 1,835 patients who died of cancer. They reported that metastatic esophageal cancer accounted for 6.1 % (112 patients) and the site of the primary tumor was as follows: lung in 51 cases, breast in 14 cases, and stomach in 13 cases. Direct invasion was noted in most of the patients who had metastatic esophageal cancer. Tongaonkar et al. [2] and Eng et al. [3] also reported that the frequency of hematogenous/lymphogenous metastasis to distant organs was only 1–1.3 %.

Renal cancer has an abundant blood supply and metastasis is frequent. It has been reported that metastasis is detected in approximately 30 % of renal cancer patients at the first hospital visit and the relative ranking of the site involved is lung > bone > regional lymph node > liver > Virchow’s node > brain [4]. However, esophageal metastasis of renal cell carcinoma is extremely rare. When we searched PubMed using keywords such as “renal cell carcinoma” and “esophageal metastasis”, we could only find 2 cases of esophageal metastasis from renal cell carcinoma. Antonio et al. [5] reported on a 62-year-old woman who underwent nephrectomy for right renal cancer 3 years before receiving transhiatal esophagectomy plus posterior mediastinal lymphadenectomy for esophageal metastasis (a polypoid tumor). She lived for 11 months postoperatively. Trentino et al. followed up on a 76-year-old woman who had esophageal metastasis of right renal cancer (a sessile polypoid tumor) at 5 years after nephrectomy, and reported that the patient survived for 10 months without surgical intervention [6].

In our patient, esophageal metastasis occurred at 10 years after right nephrectomy for renal cancer, so the time interval until metastasis was longer than in the other cases reported so far. Our renal cell carcinoma had an intermediate grade (grade 2); this is part of the reason why he had a long recurrence-free time. According to the Fuhrman classification, nuclear grade was the most significant prognostic criterion [7].

Surgical treatment of metastatic renal cancer usually has the advantage of a good performance status and prolongation of survival can be expected if the metastasis is in the lungs or adrenals [8]. According to the National Comprehensive Cancer Network Guidelines (version 2, 2011), surgical treatment is indicated when the primary tumor is in the kidney and the patient has a resectable solitary metastasis (synchronous or metachronous) [9]. Surgery was selected in our patient, because he had a resectable solitary esophageal tumor and a single mediastinal lymph node metastasis as well as a good performance status.

Metastasis of renal cancer to the esophagus is extremely rare. However, it might become more common in the future, because renal cancer is a slow growing tumor and survival is likely to become longer with improvement of therapeutic strategies. In fact, in recent years there have been occasional reports of metastasis of renal cancer to the pancreas, although this used to be rare. The 5-year survival rate after pancreatectomy for pancreatic metastasis of renal cancer was reported to be 72.6 % for 321 resected patients and 14 % for 73 non-resected patients [10]. There is a strong possibility that the incidence of both pancreatic and esophageal metastasis of renal cancer will increase in the future.

Esophageal metastasis of renal cancer can sometimes be managed. If resection is possible, endoscopic or surgical resection is the treatment of first choice. If the tumor is unresectable, multidisciplinary treatment (including molecular-targeting therapy) will be needed. In conclusion, esophageal metastasis of renal cancer is extremely rare. It will be necessary to conduct further studies in a larger number of patients to develop more appropriate treatments for esophageal metastasis of renal cancer.
